# 2122: Factors associated with unintended pregnancy in cancer survivors after cancer

**DOI:** 10.1017/cts.2017.96

**Published:** 2018-05-10

**Authors:** Lisa M. Shandley, Lauren M. Daniels, Jessica B. Spencer, Ann C. Mertens, Penelope P. Howards

## Abstract

OBJECTIVES/SPECIFIC AIMS: In the United States, it is estimated that approximately half of all pregnancies are unintended. This study examines the prevalence of unintended pregnancy in a cohort of cancer survivors and identifies factors associated with unintended pregnancy after cancer. METHODS/STUDY POPULATION: The FUCHSIA Women’s Study is a population-based study of female cancer survivors at a reproductive age of 22–45 years. Cancer survivors diagnosed between the ages of 20 and 35 years and at least 2 years postdiagnosis were recruited in collaboration with the Georgia Cancer Registry. Participants were interviewed about their reproductive histories. The prediagnosis analysis included all women who completed the interview; the postdiagnosis analysis excluded those who had a hysterectomy, bilateral oophorectomy, or tubal ligation by cancer diagnosis. RESULTS/ANTICIPATED RESULTS: Of the 1282 survivors interviewed, 57.5% reported at least 1 pregnancy before cancer diagnosis; of which, 44.5% were unintended. Of the 1088 survivors included in the postdiagnosis analysis, 36.9% reported a post-cancer pregnancy. Among those who had a pregnancy after cancer diagnosis, 38.6% reported at least 1 pregnancy was unintended. Of the 80 breast cancer survivors who had a pregnancy after diagnosis, 52.5% of them were unintended. Predictors of unintended pregnancy in cancer survivors included being younger than 30 years at diagnosis [odds ratio (OR) 2.1; 95% confidence interval (CI) 1.4, 2.9], identifying as Black (OR 1.6, 95% CI 1.1, 2.3, comparison: White), and having resumption of menses after cancer treatment (OR 8.1, 95% CI 2.0, 33.0). Compared with being <4 years from cancer diagnosis, those who were farther from diagnosis at the time of the interview also had increased odds of unintended pregnancy (4–7 years: OR 1.5, 95% CI 0.9, 2.7; 8–10 years: OR 1.3, 95% CI 0.7, 2.4; >10 years: OR 2.7, 95% CI 1.6, 4.7). DISCUSSION/SIGNIFICANCE OF IMPACT: Despite being at higher risk of infertility, cancer survivors may still be at considerable risk of unintended pregnancy. Women with certain types of cancer that are more likely to be hormone responsive, such as some types of breast cancer, may be hesitant to use hormonal birth control and thus be at higher risk of unintended pregnancy. Counseling for cancer survivors should include a discussion of the risk of unintended pregnancy and contraceptive options.

